# ICU-Acquired Weakness: Diagnostic Yield of a Clinical Diagnosis with Additional Quantification of Myosin Loss in Adult Critically Ill Patients

**DOI:** 10.3390/jcm15176698

**Published:** 2026-08-29

**Authors:** Wouter Adema, Matthias Thomas Exl, Yvette Hedström, Lars Larsson, Joerg C. Schefold, Werner J. Z’Graggen

**Affiliations:** 1 Department of Neurosurgery, Inselspital, Bern University Hospital, University of Bern, Freiburgstrasse 20, 3010 Bern, Switzerlandmatthias.exl@insel.ch (M.T.E.); 2 Department of Medical BioSciences, Radboud University Medical Center, 6525 GA Nijmegen, The Netherlands; 3 Graduate School for Health Science, University of Bern, 3012 Bern, Switzerland; 4 Department of Clinical Sciences, Swedish University of Agricultural Sciences, 750 07 Uppsala, Sweden; yvette.hedstrom@slu.se (Y.H.); lars.larsson@slu.se (L.L.); 5 Department of Intensive Care Medicine, Inselspital, Bern University Hospital, University of Bern, Freiburgstrasse 20, 3010 Bern, Switzerland; joerg.schefold@insel.ch; 6 Department of Neurology, Inselspital, Bern University Hospital, University of Bern, Freiburgstrasse 20, 3010 Bern, Switzerland

**Keywords:** critical illness, recovery of muscle weakness, outcome, morbidity, mortality, length of stay, muscle biopsy

## Abstract

**Background/Objectives:** Intensive care unit acquired weakness (ICUAW) is common in critically ill patients. However, ICUAW is an umbrella term for multiple neuromuscular pathologies, including critical illness myopathy (CIM). This study aimed to evaluate the diagnostic yield of the clinical diagnosis of ICUAW compared with that of preferential myosin loss, i.e., the hallmark of CIM. **Methods:** Critically ill patients were enrolled within 24 h of intubation. Primary outcome was the frequency of ICUAW on day 10 after ICU admission, defined by a Medical Research Council (MRC) sum score of <48. Secondary outcomes were the myosin-to-actin (M:A) ratio, strength progression and six month mortality. **Results:** A total of 46 out of 63 patients (73%) were diagnosed with ICUAW. Myosin loss correlated with muscle weakness and longer duration of mechanical ventilation. Overall, 29% had an M:A ratio ≤ 1.5 and 51% ≤ 1.7. In total, 58% of patients with an M:A ratio > 1.5 at day 10 reached the MRC sum score threshold at day 17 compared to 21% with an M:A ratio ≤ 1.5. Patients with ICUAW had a higher six-month mortality rate (24%) than patients without (0%). **Conclusions:** Absence of ICUAW at day 10 after ICU admission was related with a favourable outcome. Myosin loss with an M:A ratio ≤ 1.5 was associated with a slower recovery of muscle function.

## 1. Introduction

Neuromuscular complications are common in critically ill patients and are associated with prolonged mechanical ventilation, extended intensive care unit (ICU) and hospital stays, and increased morbidity and mortality [1,2]. Clinically, these complications often manifest as rapidly developing muscle weakness and atrophy. The proximal limb muscles and respiratory musculature are predominantly affected [3]. Within the spectrum of ICU-related neuromuscular dysfunction, the diagnosis of ICU-acquired weakness (ICUAW) is used to identify clinical neuromuscular weakness in critically ill patients [4,5].

Current clinical practice for diagnosis of ICUAW relies on determination of the Medical Research Council (MRC) sum score, assessing the voluntary strength of 12 muscle groups: three muscle groups of the upper extremity (shoulder abduction, elbow flexion, wrist extension) and three muscle groups of the lower extremity (hip flexion, knee extension, ankle dorsiflexion) of both sides. An MRC sum score below 48 is required for the diagnosis of ICUAW [6,7]. Although this classification successfully identifies clinical weakness, it serves as an umbrella term for various neuromuscular diseases and does not differentiate between disuse atrophy and an underlying neuronal or muscular pathology. This limits the prognostic value of a diagnosis of ICUAW and may hinder the standardisation of research cohorts [8].

In contrast, a diagnosis of critical illness myopathy (CIM) represents a distinct primary muscle pathology related to ICU treatment. Preferential myosin loss represents the hallmark of CIM [9,10]. Definite diagnosis of CIM requires fulfilment of several criteria, including clinical signs, neurophysiological evaluation and muscle biopsy for histological analysis [11]. Identifying CIM in the ICU setting therefore remains challenging, time-consuming and costly. For these reasons, and because of its invasiveness, muscle biopsies are rarely performed. In recent years, the biochemical determination of the myosin-to-actin ratio (M:A ratio) from small muscle punch biopsies was introduced to substitute the histological analysis, which requires larger tissue samples obtained surgically [12]. By now, no accepted threshold value for the M:A ratio exists, and different thresholds for a pathological M:A ratio have been reported, ranging from 0.9 to 1.7, with larger studies using either a threshold of 1.5 or 1.7 [12,13,14,15]. This ambiguity complicates study comparisons and leaves clinicians without a consensus.

The aim of this study was to evaluate the diagnostic yield of the clinical diagnosis of ICUAW compared with the biochemical evidence of preferential myosin loss, using different thresholds for the M:A ratio in a large representative cohort of intensive care patients.

## 2. Methods

### 2.1. Patients

Critically ill patients admitted between May 2024 and September 2025 to the ICU of the University Hospital Bern, Switzerland, were eligible for enrolment. This study is a sub-analysis of a single-centre prospective trial on long-stay critically ill patients. Inclusion criteria were aged between 18 and 80 years and receiving mechanical ventilation. Exclusion criteria were pregnancy, breastfeeding, mechanical ventilation for longer than 24 h at admission and known pre-existing polyneuropathy or myopathy, Guillain-Barré syndrome, acute or chronic spinal cord injury and diseases affecting the neuromuscular junction. Patients for whom no primary endpoint could be established on day 10 were dropped from all analyses. Eligible critically ill patients were included after an independent physician provided written informed consent. Next-of-kin were informed as soon as possible, and written informed consent was obtained once the patient’s health status permitted it. Patients were examined at admission and 10 days after ICU admission (primary endpoint). All procedures were approved by the local ethics committee (Kantonale Ethikkomission, Bern, Switzerland: project-ID 2023-01851) and complied with the Declaration of Helsinki.

### 2.2. Patient Characteristics

Baseline characteristics were established at admission for all study patients, including age, sex, BMI and admission diagnosis. The Acute Physiology and Chronic Health Evaluation II (APACHE II) score was assessed on day one, and the Sequential Organ Failure Assessment (SOFA) score was assessed on days one and 10. Additionally, on day 10, the number of hours a patient received mechanical ventilation was assessed regardless of weaning success. Length of stay (LOS) in the ICU was calculated by summing all hours the patient stayed in the ICU. LOS in the hospital was defined as all days from admission until discharge from the hospital or death and included ICU LOS. At discharge, the patient’s destination was derived from hospital records.

### 2.3. Assessment of the Medical Research Council Sum Score

Muscular strength examinations were performed on day 10 post-ICU admission or within a 48 h time window if the patient was not able to fully cooperate for the examination (Glasgow coma scale < 13). If a patient was unable to fully follow commands at any point within this time window, the best MRC sum score achieved during that period was used. The muscle strength was evaluated bilaterally according to the MRC sum score for three functional muscle groups on the upper extremities (shoulder abduction, elbow flexion, and wrist extension) and three on the lower extremities (hip flexion, knee extension, and ankle dorsiflexion) [16]. Individual muscle groups were scored from zero to five, summing a maximum score of 60 points. In patients with central motor deficits, the unaffected side was assessed and mirrored for the final MRC sum score. ICUAW was defined as an overall MRC sum score of less than 48 points [6,7]. This 48 h time window was chosen in order to assess each patient’s best possible motor performance, despite the sometimes fluctuating levels of alertness and cooperation typical of this stage of critical illness. The MRC sum score was reassessed at day 17 after admission to the ICU.

### 2.4. Fine-Needle Muscle Biopsy and Measurement of the M:A Ratio

On day 10 post-admission, a fine-needle punch biopsy of the tibialis anterior muscle was performed to measure the M:A ratio, using a 16-gauge soft-tissue, semi-automated, disposable biopsy needle (Temno Evolution^®^, Merit Medical, South Jordan, UT, USA). Samples were immediately frozen (−80 °C). The detailed methodology of the analysis has been described previously [12]. Briefly, myosin and actin quantification was determined by 12% SDS-PAGE using approximately half of the biopsy. The gels were stained using SimplyBlue SafeStain (Invitrogen, Carlsbad, CA, USA) and subsequently scanned in a GS-900 calibrated densitometer (Bio Rad, Hercules, CA, USA). The volume integration function was used to quantify the amount of protein on 12% and 6% gels (ImageQuant TL Software version 3.3, Amersham Biosciences, Uppsala, Sweden).

### 2.5. Groups

Patients with an MRC sum score of 48 or higher at day 10 were defined as the non-ICUAW group. For patients with ICUAW (MRC sum score < 48 points), a preferential loss of myosin filaments indicative of CIM was evaluated through two pre-specified exploratory analyses using the two M:A ratio thresholds that were most frequently used in prior larger studies (12–15). These two M:A ratio thresholds were selected a priori. For each analysis, the ICUAW cohort was subdivided into mutually exclusive groups with corresponding thresholds of > or ≤1.5 and > or ≤1.7.

### 2.6. Outcome Measures

The primary endpoint was the frequency of ICUAW in the entire cohort. Secondary endpoints were frequencies of preferential myosin loss indicative of CIM for an M:A ratio threshold ≤1.5 and ≤1.7, the association between M:A ratio and MRC sum score and between M:A ratio and hours of mechanical ventilation, strength progression within one week and mortality rates. The rate of strength progression was assessed binarily: whether patients reached the threshold MRC sum score of ≥48 at day 17. Mortality rates were accessible through hospital records and actively monitored at one, three and six months after ICU admission.

### 2.7. Statistical Analysis

Statistical analyses were performed using Python (version 3.13). Baseline characteristics are presented descriptively as means (±SD) or medians [IQR] and frequencies as counts with percentages. Normality was assessed using Shapiro–Wilk tests. Although the Shapiro–Wilk test indicated a significant deviation from normality for the APACHE II score, inspection of the corresponding Q-Q plot and histogram showed an approximately normal distribution without marked skewness or outliers; parametric testing was therefore retained for this variable, while it is reported descriptively as median [IQR] consistent with non-normally distributed variables. In line with reporting guidelines for observational data, formal statistical testing was not performed for baseline characteristics to prioritise clarity and readability.

Numerical baseline variables of the non-ICUAW group were compared to patients with ICUAW subdivided based on the two M:A ratio thresholds using separate one-way ANOVAs with Bonferroni correction for multiple testing. Pearson’s correlation analyses were performed to assess linear associations between the M:A ratio and the MRC sum score as well as the M:A ratio and hours of mechanical ventilation using correlation coefficients (r) and *p*-values.

Proportions of patients in the different groups reaching or surpassing the threshold MRC sum score of ≥48 points on day 17 were compared with a complete-case analysis using Fisher’s exact test. Sensitivity and specificity analyses were performed in patients with ICUAW on day 17, using M:A ratio thresholds of ≤1.5 and ≤1.7 as the test-positive results and persistent weakness (failure to reach an MRC sum score of ≥48 at day 17) as the positive outcome. Patients for whom no MRC sum score could be established on day 17 were removed from this analysis. Survival distributions were compared between groups with an omnibus log-rank test for each M:A ratio threshold. All statistical tests were two-sided, and a *p*-value < 0.05 was considered statistically significant. Given the pre-specified exploratory nature of the M:A ratio threshold analyses, Fisher’s exact tests and log-rank tests were not corrected for multiple comparisons and should be interpreted as hypothesis-generating; by contrast, Bonferroni correction was applied to the ANOVA post hoc comparisons of baseline characteristics to control for multiplicity across the four subgroups.

## 3. Results

A total of 102 critically ill patients were enrolled. Of these, 35 patients did not reach the primary endpoint and dropped out (patients died (n = 15); patients were transferred to another hospital (n = 12); and patients withdrew their consent (n = 8)). Additionally, three patients were excluded due to missing MRC sum scores at day 10 and one due to a missing M:A ratio. The final analysis included 63 patients, of whom 46 (73%) had an MRC sum score < 48 on day 10 and therefore fulfilled the diagnosis of ICUAW, while 17 patients (27%) had an MRC sum score ≥ 48 (non-ICUAW group). Of all patients fulfilling the diagnosis of ICUAW, 18 (39%) had an M:A ratio ≤ 1.5 and 32 (70%) ≤ 1.7. There was no M:A ratio ≤ 1.5 observed in the non-ICUAW group. Five patients in the non-ICUAW group had an M:A ratio ≤ 1.7. Compared with the 63 patients included in the final analysis (mean age 58.2 years, 67% male, median APACHE II 29.0 [25.0–34.0]), the 39 excluded patients were slightly older (mean age 61.6 years), had a similar sex distribution (62% male), and had a higher illness severity at admission (median APACHE II 33 [29–37]).

Baseline characteristics of the entire cohort included in the final analysis, stratified based on the presence of ICUAW, are presented in Table 1.

Patients with ICUAW are further stratified according to the M:A ratio thresholds > or ≤1.5 and > or ≤1.7 (Table 2). Corresponding statistical comparisons are shown in Table 3 and Table 4. At admission, the non-ICUAW group demonstrated lower baseline disease severity, characterised by lower APACHE II (*p* = 0.004) and SOFA score on day 1 (*p* = 0.012). Furthermore, the non-ICUAW group showed a lower SOFA score on day 10 and a shorter duration of mechanical ventilation compared to patients with ICUAW. Notably, these differences between the non-ICUAW group and patients with ICUAW were not affected by the M:A ratio threshold, except for the APACHE II score, which was not statistically different between the non-ICUAW group and patients with an M:A ratio ≤ 1.7.

**Figure 1:**
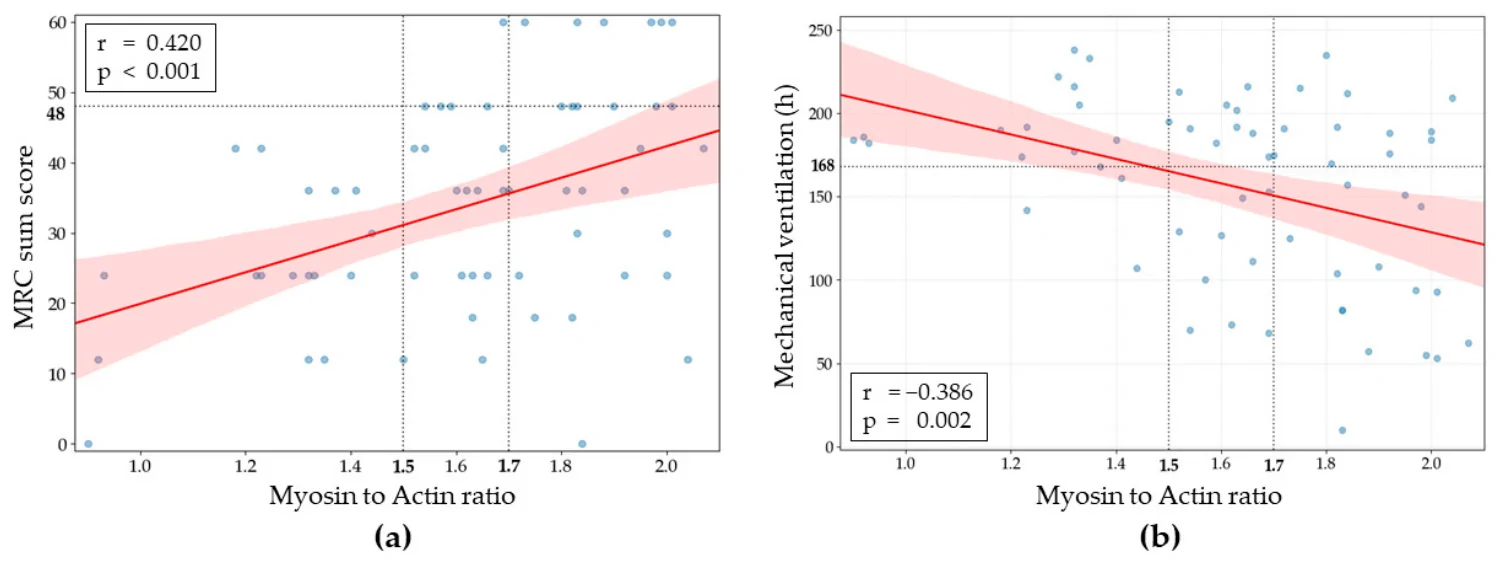
(**a**) Scatterplot illustrating the relationship between the myosin-to-actin (M:A) ratio and the Medical Research Council (MRC) sum score. The red line represents the linear regression fit, with the shaded red area indication the 95% confidence interval. The horizontal dashed line at the MRC sum score of 48 indicates the diagnostic threshold for intensive care unit acquired weakness. Vertical dashed lines indicate the M:A ratio threshold at 1.5 and 1.7. (**b**) Scatterplot illustrating the relationship between the M:A ratio and the number of hours of mechanical ventilation. The red line represents the linear regression fit, with the shaded red area indication the 95% confidence interval. The horizontal line at 168 h indicates seven days of consecutive mechanical ventilation.

The M:A ratio correlated positively with the MRC sum score (r = 0.420, *p* < 0.001) and negatively with the hours of mechanical ventilation (r = −0.386, *p* = 0.002) (Figure 1).

**Figure 2:**
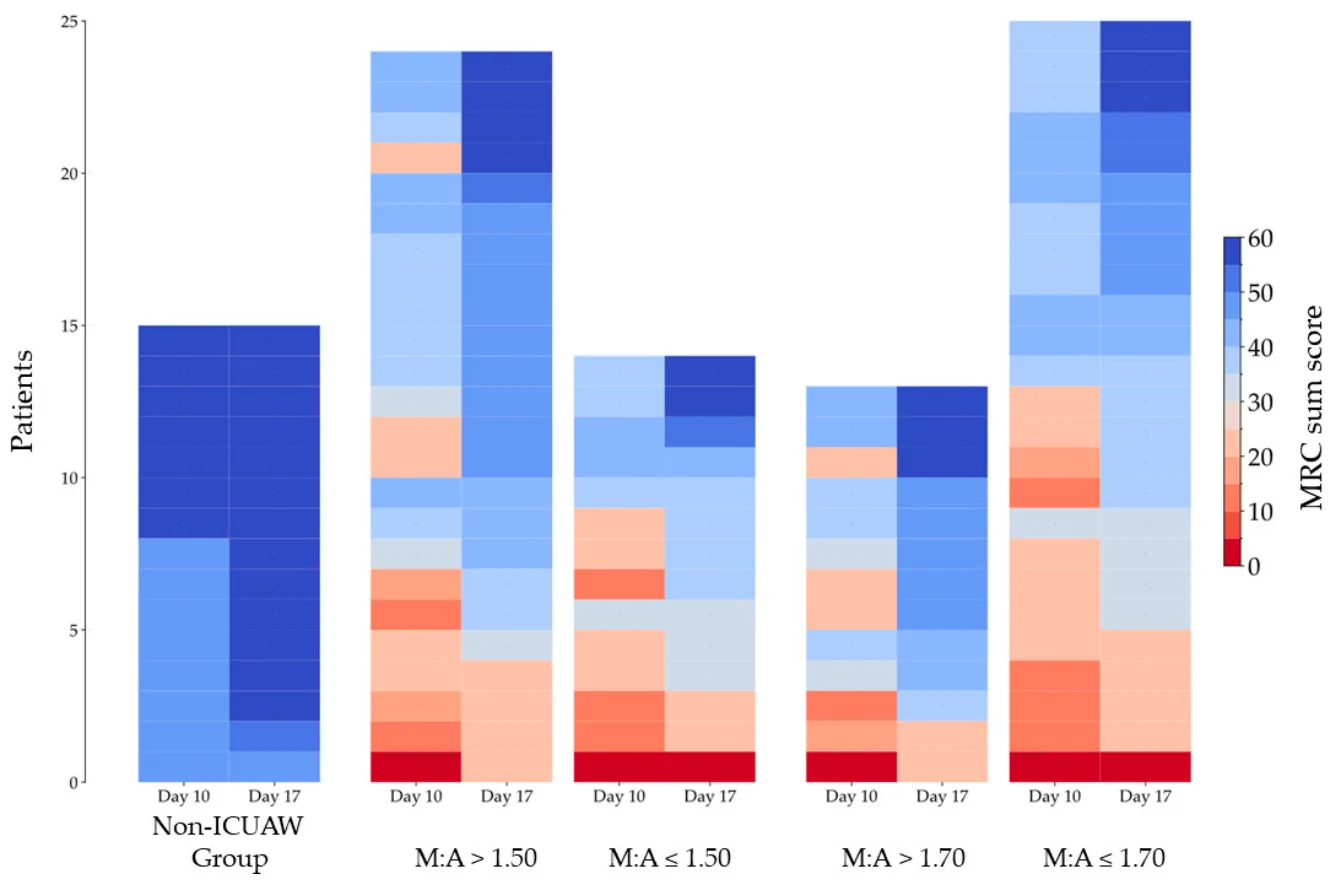
Heatmaps illustrating the Medical Research Council (MRC) sum score of individual patients at days 10 and 17. The columns represent the non-ICUAW group and patients with intensive care unit acquired weakness stratified by myosin-to-actin (M:A) ratio thresholds. Colour intensity scales from red (lower MRC sum scores) to blue (higher MRC sum scores), as shown in the step-legend on the right.

Strength progression between day 10 and day 17 after ICU admission could be established in 53 patients and is illustrated using heatmaps in Figure 2. Five patients were excluded from this analysis due to missing MRC sum scores on day 17, and five due to death between days 10 and 17. No patients had a lower MRC sum score at day 17 compared to day 10. Proportions of patients reaching the ICUAW threshold score of ≥48 are shown in Table 5, separately for the same M:A ratio subgroups as in Table 2. Fourteen out of 24 patients with an M:A ratio > 1.5 at day 10 reached the MRC sum score threshold ≥48 at day 17, whereas only three out of 14 patients with an M:A ratio ≤ 1.5 reached this threshold (Fisher’s exact test *p* = 0.043). In contrast, for the M:A ratio threshold > or ≤1.7, there was no statistical significance in MRC sum score progression between the groups (*p* = 0.178). In the sensitivity and specificity analyses, using an M:A ratio ≤ 1.5 or ≤1.7 as the test-positive result for persistent clinical weakness up to day 17, we observed a 52% sensitivity and 82% specificity with the ≤1.5 threshold and 76% sensitivity and 47% specificity with the ≤1.7 threshold.

**Figure 3:**
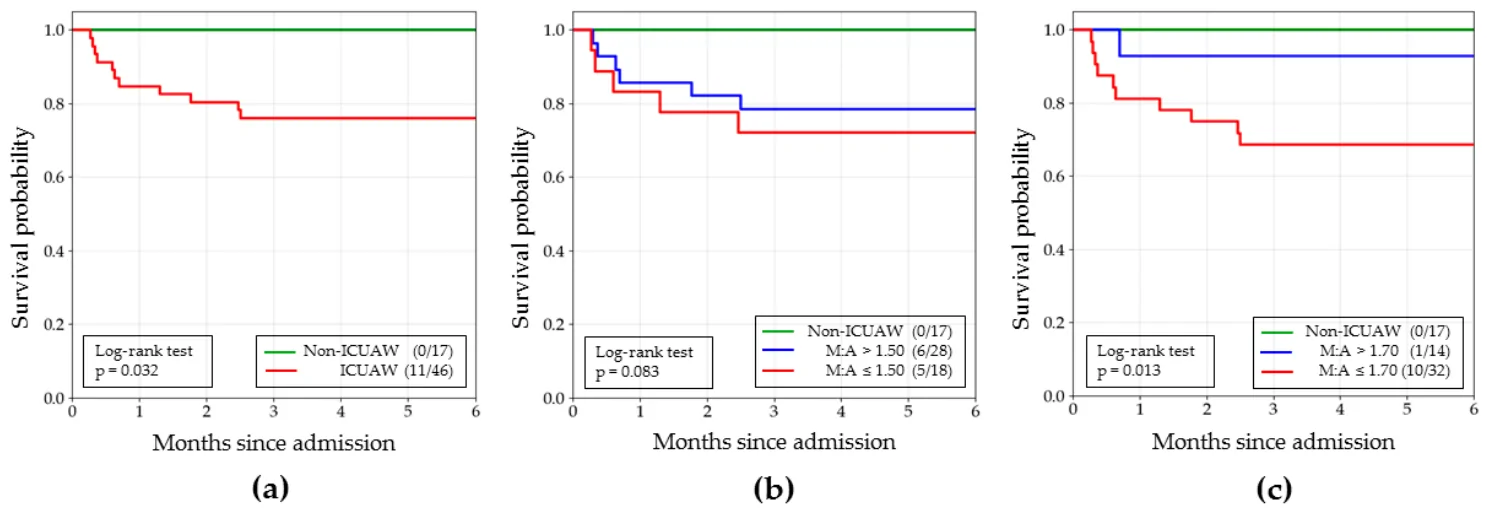
Six-month survival curves: (**a**) patients with intensive care unit acquired weakness (ICUAW) and the non-ICUAW group; (**b**) patients with a myosin-to-actin (M:A) ratio > or ≤1.5 and the non-ICUAW group; (**c**) patients with an M:A ratio > or ≤1.7 and the non-ICUAW group.

Patients with ICUAW had a higher mortality rate than the non-ICUAW group six months post-ICU admission (log-rank test *p* = 0.032). The mortality rate in patients with an M:A ratio > 1.5 was 21.4% compared to 27.8% in patients with an M:A ratio ≤ 1.5 (*p* = 0.083) and 7.1% in patients with an M:A ratio > 1.7 versus 31.3% in patients with an M:A ratio ≤ 1.7 (*p* = 0.013) (Figure 3). There were no deaths observed in the non-ICUAW group.

Legend fractions indicate deaths and the corresponding group size. Overall differences between the curves in panel A were analysed using a log-rank test and in panels B and C using an omnibus log-rank test.

## 4. Discussion

In this prospective single-centre cohort study in critically ill patients, the frequency of ICUAW was as high as 73%. With preferential myosin loss indicative of CIM used as an additional criterion, the frequency fell depending on the M:A ratio threshold: 29% of all patients (39% with ICUAW) had an M:A ratio ≤ 1.5, and 51% of all patients (70% with ICUAW) had an M:A ratio ≤ 1.7. While the absence of ICUAW at day 10 after ICU admission was associated with a favourable outcome, preferential myosin loss with an M:A ratio threshold ≤1.5 was indicative of slower recovery of muscle function and a longer need for mechanical ventilation. In contrast, an M:A ratio threshold ≤1.7 appeared to discriminate mortality better than the ≤1.5 threshold.

The frequency of ICUAW of 73% in our cohort corresponds to earlier reported frequencies, although these vary considerably depending on the ICU setting and study population. In a homogeneous cohort of critically ill COVID-19 patients, frequencies between 72 and 92% were reported [8,17]. In less selective cohorts, comparable with ours, reported ICUAW frequencies were slightly lower, ranging from 59 to 65% [18,19]. In a pooled analysis including critically ill patients ventilated for ≥7 days, the observed mean frequency of ICUAW was 43% (interquartile range 25–75%) [20]. This high variability of reported ICUAW frequencies can be attributed to several factors, e.g., the studied patient cohort, timepoint of clinical examination and patients’ ability to follow the examination, medication, neurological conditions that affect the examination (i.e., disease of the central nervous system), and others.

Patients who did not develop ICUAW in our cohort were less severely ill as observed by lower APACHE II and SOFA scores at ICU admission, compared to patients who were diagnosed with ICUAW. But, more importantly, the absence of ICUAW at day 10 after ICU admission was paralleled by a six-month mortality rate of zero, as well as a shorter need for mechanical ventilation and a shorter LOS in the ICU. Hence, the absence of ICUAW at day 10 after ICU admission seems to be a good discriminator for a favourable outcome. In other words, excluding a neuromuscular complication of ICU treatment by pure clinical assessment of the MRC sum score in the subacute phase of ICU treatment can be used as an estimate for a more benign ICU outcome. This is well in line with earlier reports [21,22,23,24].

There was a positive correlation between myosin loss, as determined by the M:A ratio, and muscle weakness assessed by the MRC sum score (Figure 1a). Furthermore, duration of mechanical ventilation showed a negative correlation with the M:A ratio, which may partly reflect diaphragm weakness in addition to disease severity. These findings support an association between myosin loss and muscle strength in critically ill patients. This is consistent with a recent prospective study using repeated muscle biopsies, which demonstrated that acute muscle weakness in critically ill patients was associated with a loss of myosin rather than atrophy [25].

Assessing myosin loss in critically ill patients, in addition to the purely clinical diagnosis of ICUAW, may help identify a muscular contribution to weakness [26]. Published M:A ratio threshold values used for diagnosis of CIM vary in the literature, with the largest studies using either 1.5 or 1.7 [12,14,27]. Setting the threshold of the M:A ratio at 1.5 identified 29% of all patients and 39% of patients with ICUAW, with myosin loss indicative of CIM in our cohort. This corresponds with the reported frequencies of definite CIM of 31% by Derde et al. (2012) but is lower than the reported 55% by Rodriguez et al. (2022) in a more severely ill homogeneous cohort of COVID-19 patients with acute respiratory distress syndrome [14,27]; however, both studies established CIM using full diagnostic criteria (clinical, electrophysiological, and histological), whereas our study assessed the M:A ratio alone, limiting the direct comparability of these frequencies.

The higher M:A ratio threshold of ≤1.7 resulted in an expected increase in the number of patients attributed to myosin loss indicative of CIM to 51%, approaching the 55% definite CIM frequency reported in the more severely ill cohort described by Rodriguez et al. (2022) [27], despite the difference in diagnostic methodology noted above. In addition, five patients without ICUAW showed an M:A ratio ≤ 1.7. One possible interpretation could be that these patients displayed a subclinical myosin loss causing a not-assessable clinical weakness at the time of evaluation.

Since none of these patients showed a decrease in the MRC sum score between days 10 and 17, it is less likely that the reduced M:A ratio was caused by an evolving muscular affection at the timepoint of evaluation than by an already improving one or a clinically not-expressed one. Furthermore, the M:A ratio threshold ≤1.5 appeared to identify patients with a slower recovery of muscle weakness more accurately (52% sensitivity and 82% specificity) than the higher threshold of ≤1.7 (76% sensitivity and 47% specificity) or the pure clinical diagnosis of ICUAW (Table 5, Figure 2). However, given the small subgroup sizes (n = 13/32), these comparisons are possibly underpowered and should be considered as hypothesis-generating rather than confirmatory. In summary, the additional determination of the M:A ratio may help identify both critically ill patients with a muscular component of weakness and using the threshold of ≤1.5, critically ill patients with a slower recovery of muscle function, a cohort that benefits most from future CIM research. An M:A ratio threshold of ≤1.7 appears, from a purely clinical point of view, less specific as it also captures critically ill patients without ICUAW/relevant muscular weakness. Unexpectedly, cases of mortality could be better discriminated using the less selective M:A ratio threshold of ≤1.7 or the clinical diagnosis of ICUAW than with the M:A ratio threshold of ≤1.5. This might be due to the fact that mortality is more closely related to overall disease severity than to myosin loss indicative of CIM, which itself depends on disease severity as already elaborated above.

## 5. Conclusions

ICUAW is prevalent among mechanically ventilated critically ill patients who survive to day 10 and are able to complete clinical assessment. In this population, our results suggest that the absence of ICUAW at day 10 after ICU admission is associated with a more favourable outcome and that preferential myosin loss is associated with both muscle weakness and longer duration of mechanical ventilation. In addition, an M:A ratio threshold of ≤1.5 was associated with a slower recovery of muscle function in this cohort of critically ill patients. These associations should be interpreted with caution, as our analyses were largely unadjusted for potential confounders such as disease severity.

### Limitations

Some important limitations deserve discussion. Although this study was prospectively conducted in a large tertiary university hospital in a cohort of adult critically ill patients with a wide heterogeneity of diseases, the single-centre study design might cause some bias as well as the selection of patients, which may limit overall generalisability. Furthermore, as expected, there was a high drop-out rate of patients. These patients were either severely ill (patients died) or with a favourable disease course (e.g., patients transferred to another hospital). Hence, we cannot exclude that the reported frequency of ICUAW is affected by these factors. While the MRC sum score was assessed within a 48 h time window centred on day 10 to ensure optimal alertness and cooperation, the invasive muscle biopsy was performed in all patients standardised according to the study protocol on day 10. This resulted in a time lag between these two assessments in seven patients. Overall, we consider this time lag as less important for the study outcome than the risk of an inadequate assessed MRC sum score. Importantly, our analyses of the associations between the M:A ratio, MRC sum score, duration of mechanical ventilation, and mortality were largely unadjusted; as these measures, with the exception of the M:A ratio, may be influenced by overall disease severity, these findings should be interpreted with caution and as associations rather than causal or mechanistic relationships. We also restricted our analyses to the M:A ratio thresholds of 1.5 and 1.7, as these are used by the largest studies published to date; lower thresholds (0.9–1.4), reported in smaller, earlier studies, were not additionally tested. The diagnostic criteria for CIM by Latronico and Bolton require, in addition to the clinical examination and determination of myosin loss [11], a comprehensive neurophysiological examination, which was beyond the scope of this study. Nevertheless, the loss of myosin, quantified based on the M:A ratio, can be used as a marker of muscle involvement and ultimately also as an indicator for CIM. There was an imbalance in sex in the ICUAW group. Given known sex differences in muscle composition and myosin turnover, we cannot exclude that sex may act as a confounder, especially in the observed association between M:A ratio and MRC sum score. Because of the available sample size, we had to omit a sex-stratified analysis.

## Figures and Tables

**Table 1:** Patient characteristics.

	All Patients (N = 63)	Non-ICUAW Group (n = 17)	ICUAW (n = 46)
**At admission**			
Age (min–max)	58.2 (28–79)	52.6 (29–76)	60.3 (28–79)
Sex (female/male)	21/42	9/8	12/34
BMI (kg/m^2^)	26.7 (±4.9)	24.3 (±3.1)	27.6 (±5.1)
APACHE II	29.0 [25.0–34.0]	27.0 [22.0–29.0]	30.0 [27.0–35.0]
SOFA (day 1)	11.2 (±3.4)	9.4 (±2.3)	11.8 (±3.6)
SOFA (day 10)	7.5 (±4.3)	4.3 (±2.4)	8.7 (±4.2)
Mechanical ventilation (h)	155.7 (±54.8)	104.5 (±53.4)	174.7 (±42.0)
Length of stay in ICU (days)	19.4 (±16.3)	12.2 (±6.3)	22.1 (±18.0)
Length of stay in hospital (days)	31.7 (±26.3)	25.8 (±18.2)	33.9 (±28.5)
**Diagnosis at admission**			
Neuro	20 (32%)	11 (65%)	9 (20%)
Sepsis	15 (24%)	0	15 (33%)
Heart failure	14 (22%)	2 (12%)	12 (26%)
Trauma	9 (14%)	2 (12%)	7 (15%)
ARDS	4 (6%)	1 (6%)	3 (7%)
Other	1 (2%)	1 (6%)	0
**Discharge destination**			
Rehabilitation	32 (51%)	8 (47%)	24 (52%)
Transferred to another hospital	18 (29%)	4 (24%)	14 (30%)
Patient died in the hospital	8 (13%)	0	8 (17%)
Home	4 (6%)	4 (24%)	0
Other	1 (2%)	1 (6%)	0

Note: Patient characteristics of all patients and patients fulfilling or not fulfilling the diagnosis of intensive care unit acquired weakness (ICUAW) presented as mean (±standard deviation) or median [interquartile range]. The diagnoses are reported as counts and percentages. Body mass index (BMI); acute physiology and chronic health evaluation II score (APACHE II); sequential organ failure assessment score (SOFA); intensive care unit (ICU); and acute respiratory distress syndrome (ARDS).

**Table 2:** Characteristics of patients with ICUAW stratified by M:A ratios.

	M:A > 1.50 (n = 28)	M:A ≤ 1.50 (n = 18)	M:A > 1.70 (n = 14)	M:A ≤ 1.70 (n = 32)
**At admission**				
Age (min–max)	60.5 (28–79)	60.1 (39–79)	62.8 (28–77)	59.2 (37–79)
Sex (female/male)	5/23	7/11	2/12	10/22
BMI (kg/m^2^)	27.7 (±5.7)	27.6 (±4.2)	29.1 (±5.6)	27.0 (±4.8)
APACHE II	31.0 [26.5–35.0]	29.5 [27.3–35.5]	33.0 [29.3–35.5]	29.0 [25.0–35.0]
SOFA (day 1)	11.4 (±3.2)	12.6 (±4.0)	12.3 (±2.9)	11.6 (±3.8)
SOFA (day 10)	7.9 (±3.8)	9.8 (±4.7)	8.6 (±3.2)	8.7 (±4.6)
Mechanical ventilation (h)	167.1 (±46.4)	186.4 (±31.6)	169.9 (±45.6)	176.8 (±40.8)
Length of stay in ICU (days)	24.4 (±22.0)	18.4 (±8.5)	23.8 (±23.5)	21.3 (±15.5)
Length of stay in hospital (days)	38.7 (±33.9)	26.3 (±15.2)	40.5 (±28.6)	31.0 (±28.5)
**Diagnosis at admission**				
Neuro	7 (25%)	2 (11%)	3 (21%)	6 (19%)
Sepsis	7 (25%)	8 (44%)	3 (21%)	12 (38%)
Heart failure	7 (25%)	5 (28%)	5 (36%)	7 (22%)
Trauma	5 (18%)	2 (11%)	2 (14%)	5 (16%)
ARDS	2 (7%)	1 (6%)	1 (7%)	2 (6%)
**Discharge destination**				
Rehabilitation	17 (61%)	7 (39%)	10 (72%)	14 (44%)
Transferred to another hospital	7 (25%)	7 (39%)	3 (21%)	11 (34%)
Patient died in the hospital	4 (14%)	4 (22%)	1 (7%)	7 (22%)

Note: Comparison of patients based on myosin-to-actin (M:A) ratio presented as mean (±standard deviation) or median [interquartile range]. The diagnoses are reported as counts and percentages. Body mass index (BMI); acute physiology and chronic health evaluation II score (APACHE II); sequential organ failure assessment score (SOFA); intensive care unit (ICU); and acute respiratory distress syndrome (ARDS).

**Table 3:** Comparison of the non-ICUAW group and patients with ICUAW stratified by M:A ratios.

	One-Way ANOVA				Post Hoc Analysis		
	DFn	DFd	F	*p*	Non-ICUAW Group vs. M:A > 1.50	Non-ICUAW Group vs. M:A ≤ 1.50	M:A > 1.50 vs. M:A ≤ 1.50
**At admission**							
Age	2	60	2.175	0.123	*p* = 0.238	*p* = 0.377	*p* = 1.0
BMI (kg/m^2^)	2	60	3.210	0.047	*p* = 0.038	*p* = 0.036	*p* = 1.0
APACHE II	2	60	4.558	0.014	*p* = 0.026	*p* = 0.045	*p* = 1.0
SOFA (day 1)	2	60	4.123	0.021	*p* = 0.069	*p* = 0.024	*p* = 0.889
SOFA (day 10)	2	60	9.678	<0.001	*p* = 0.001	*p* < 0.001	*p* = 0.454
Mechanical ventilation (h)	2	60	16.216	<0.001	*p* = 0.001	*p* < 0.001	*p* = 0.302
Length of stay in ICU (days)	2	60	3.254	0.046	*p* = 0.027	*p* = 0.059	*p* = 0.584
Length of stay in hospital (days)	2	60	1.855	0.165	*p* = 0.315	*p* = 1.0	*p* = 0.295

Note: Comparison of the non-ICUAW group (n = 17) with patients subdivided based on a myosin-to-actin (M:A) ratio > 1.50 (n = 28) or ≤1.50 (n = 18). Groups were compared using a one-way ANOVA. Significant main effects were followed up with pairwise post hoc tests using the Bonferroni correction for multiple comparisons to adjust the significance level. Body mass index (BMI); acute physiology and chronic health evaluation II score (APACHE II); sequential organ failure assessment score (SOFA); and intensive care unit (ICU).

**Table 4:** Comparison of the non-ICUAW group and patients with ICUAW stratified by M:A ratios.

	One-Way ANOVA				Post Hoc Analysis		
	DFn	DFd	F	*p*	Non-ICUAW Group vs. M:A > 1.70	Non-ICUAW Group vs. M:A ≤ 1.70	M:A > 1.70 vs. M:A ≤ 1.70
**At admission**							
Age	2	60	2.556	0.086	*p* = 0.134	*p* = 0.395	*p* = 1.0
BMI (kg/m^2^)	2	60	4.337	0.017	*p* = 0.028	*p* = 0.060	*p* = 0.699
APACHE II	2	60	5.434	0.007	*p* = 0.006	*p* = 0.061	*p* = 0.582
SOFA (day 1)	2	60	3.522	0.036	*p* = 0.019	*p* = 0.044	*p* = 1.0
SOFA (day 10)	2	60	7.864	0.001	*p* = 0.001	*p* < 0.001	*p* = 1.0
Mechanical ventilation (h)	2	60	14.875	<0.001	*p* = 0.003	*p* < 0.001	*p* = 1.0
Length of stay in ICU (days)	2	60	2.494	0.091	*p* = 0.283	*p* = 0.017	*p* = 1.0
Length of stay in hospital (days)	2	60	1.226	0.301	*p* = 0.336	*p* = 1.0	*p* = 0.929

Note: Comparison of the non-ICUAW group (n = 17) with patients subdivided based on a myosin-to-actin (M:A) ratio > 1.70 (n = 14) or ≤1.70 (n = 32). Groups were compared using a one-way ANOVA. Significant main effects were followed up with pairwise post hoc tests using the Bonferroni correction for multiple comparisons to adjust the significance level. Body mass index (BMI); acute physiology and chronic health evaluation II score (APACHE II); sequential organ failure assessment score (SOFA); and intensive care unit (ICU).

**Table 5:** Progression of MRC sum scores between day 10 and 17 stratified by M:A ratio.

	M:A Ratio	MRC Sum Score (n = 63)	MRC Sum Score (n = 53)	Surpassed ICUAW Threshold
		Day 10	Day 17	
**Non-ICUAW Group** n = 17		53.6 (±6.20)	58.8 (±3.36)	15/15 (100%)
**ICUAW** n = 46		27.8 (±11.87)	40.7 (±13.75)	17/38 (45%)
	>1.50 n = 28	29.3 (±11.37)	43.8 (±11.80)	14/24 (58%)
**ICUAW**				
	≤1.50 n = 18	25.3 (±12.71)	35.6 (±15.69)	3/14 (21%)
	>1.70 n = 14	27.2 (±12.15)	45.2 (±11.90)	8/13 (62%)
**ICUAW**				
	≤1.70 n = 32	28.1 (±11.96)	38.4 (±14.28)	9/25 (36%)

Note: Values represent mean (±standard deviation). The last column indicates the number and percentage of participants within each subgroup who achieved or surpassed the ICUAW recovery threshold (MRC sum score ≥ 48) by day 17. Myosin-to-actin (M:A) ratio; Medical Research Council (MRC) sum score; and intensive care unit acquired weakness (ICUAW).

## Data Availability

The data that support the findings of this study are available from the corresponding author upon reasonable request.
